# Synthesis of Aryliron Complexes [CpFe(CO)_2_Ar] by Palladium-Catalyzed Reactions of [CpFe(CO)_2_I] with Arylzinc, -Boron, or -Indium Reagents

**DOI:** 10.3390/ma2030978

**Published:** 2009-08-20

**Authors:** Shigeo Yasuda, Yoshihiro Asada, Hideki Yorimitsu, Koichiro Oshima

**Affiliations:** Department of Material Chemistry, Graduate School of Engineering, Kyoto University, Kyoto-daigaku Katsura, Nishikyo-ku, Kyoto 615-8510, Japan

**Keywords:** iron, aryl metal, transmetalation, palladium, zinc, indium, boron

## Abstract

Transmetalation between [CpFe(CO)_2_I] and arylzinc iodide-lithium chloride complexes proceeds in the presence of catalytic amounts of palladium acetate and *N*,*N*,*N’*,*N’*-tetramethyl-1,2-cyclohexanediamine to yield the corresponding aryliron complexes [CpFe(CO)_2_Ar]. Phenylation of [CpFe(CO)_2_I] also takes place when triphenylindium is used under similar conditions. Arylboronic acids undergo arylation in the presence of cesium carbonate and a palladium-*N*-heterocyclic carbene complex, PEPPSI.  The present methods are useful for the facile synthesis of various functionalized [CpFe(CO)_2_Ar]. The products [CpFe(CO)_2_Ar] represent an interesting class of aryl metals that undergo several transformation.

## 1. Introduction

Aryldicarbonylcyclopentadienyliron complexes [CpFe(CO)_2_Ar] are important as typical 18-electron organometallics [1], reagents in organic synthesis [2], and functional organic materials [3,4,5]. Despite their importance, there had been few reports of a concise and general synthesis of [CpFe(CO)_2_Ar] [6,7,8,9,10]. Recently we reported catalytic reactions for the synthesis of [CpFe(CO)_2_Ar]. Our first report showed that transmetalation between [CpFe(CO)_2_I] and arylmagnesium reagents proceeds smoothly under palladium catalysis [11]. However, the scope of the reaction is limited due to the high reactivity of arylmagnesium reagents. Subsequently, we reported the use of arylzinc or arylboron reagents for palladium-catalyzed arylation reactions of [CpFe(CO)_2_I], taking advantage of the mild reactivities of these reagents [12]. The reactions with arylzinc or arylboron reagents showed excellent functional group compatibility and allowed us to prepare a wide range of [CpFe(CO)_2_Ar]. Here we report the full details of the improved method and the utility of the products.

## 2. Results and Discussion 

### 2.1. Reactions of [CpFe(CO)_2_I] with Arylzinc Reagents

We initially examined the reaction of [CpFe(CO)_2_I] (**1**) with commercially available PhZnI (Aldrich) in the presence of 0.25 mol% of palladium acetate and 0.50 mol% of *trans*-*N*,*N*,*N’*,*N’*-tetra-methyl-1,2-cyclohexanediamine (Table 1, entry 1). The reaction proceeded to yield the corresponding product **2a** in only 46% yield. A 29% of starting material **1** was recovered, and dimer **3** was detected as the only identifiable byproduct. We then tried to use the more reactive arylzinc reagent reported by Knochel [13]. Treatment of iodobenzene with zinc powder in the presence of lithium chloride at 50 °C for 24 h afforded PhZnI•LiCl. The reaction with PhZnI•LiCl was indeed successful, affording **2a** quantitatively (90% isolated yield) (entry 2). It is worth noting that the reaction of **1** with PhZnI•LiCl proceeded less efficiently in the absence of the palladium catalyst (25 °C, 30 min) to provide **2a** in only 52% yield. 

**Table 1:**
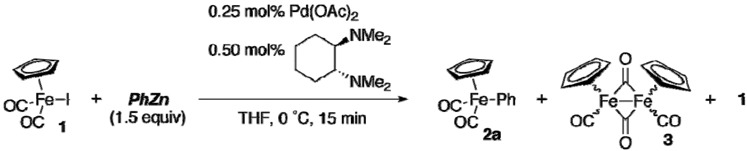
Reactions of [CpFe(CO)_2_I] (**1**) with Several Types of Phenylzinc Reagents.

Entry	*PhZn*	Yield based on ^1^H-NMR /%		
		2a	3	1
1	PhZnI	46	15	29
2	PhZnI • LiCl	100 (90)	0	0
3	PhZnI + LiCl	20	34	35
4	PhZnI + LiCl then 50 ˚C, 24 h	73	0	17
5	ZnCl_2_ + 2 PhMgBr	100	0	0

The use of Knochel’s arylzinc reagent was indispensable. The yield of **2a** was low when we used a phenylzinc reagent prepared by mixing commercially supplied LiCl-free phenylzinc iodide and LiCl just prior to use (entry 3). The complexation of PhZnI with LiCl would not proceed efficiently at ambient temperature, but it proceeded to near completion upon heating a mixture of PhZnI and LiCl at 50 °C for 24 h to ensure sufficient reactivity (entry 4). The use of diphenylzinc prepared from ZnCl_2_ and 2 equiv of PhMgBr was also effective (entry 5), although we could not prepare functionalized diarylzinc by this method. The scope of arylzinc reagents is wide, as shown in Table 2. Although the reaction of **1** with sterically demanding aryl Grignard reagent had failed previously [11], the present method allowed us to introduce aryl groups bearing a substituent at the 2 position (entries 2, 3, and 6). The electronic nature of arylzinc reagents had little effect on the yield of arylirons (entries 4–10). The modest reactivity of organozinc reagents opened the way for facile preparation of aryliron complexes having bromo, cyano, and ethoxycarbonyl groups (entries 7–10). However, the reaction with 2-ethoxycarbonylphenylzinc reagent or 4-acetylphenylzinc reagent resulted in recovery of **1** (entries 11 and 12). Thienyliron complex **2m** was obtained in high yield, while no reaction took place with 3-pyridylzinc reagent (entries 13 and 14).

**Table 2:**
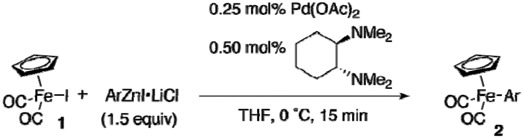
Palladium-Catalyzed Arylation of [CpFe(CO)_2_I] with Arylzinc Reagents ArZnI•LiCl.

Entry	Ar	2	Isolated yield /%
1	Ph	**2a**	90
2	1-naphthyl	**2b**	92
3	2-MeC_6_H_4_	**2c**	73^a^
4	4-MeOC_6_H_4_	**2d**	94
5	3-CF_3_C_6_H_4_	**2e**	94
6	2-FC_6_H_4_	**2f**	74
7	4-BrC_6_H_4_	**2g**	92
8	4-NCC_6_H_4_	**2h**	94
9	4-EtOC(=O)C_6_H_4_	**2i**	82
10	3-EtOC(=O)C_6_H_4_	**2j**	90
11	2-EtOC(=O)C_6_H_4_	**2k**	trace
12	4-MeC(=O)C_6_H_4_	**2l**	trace
13	2-thienyl	**2m**	79
14	3-pyridyl	**2n**	trace

^a^ 3.0 equiv of the zinc reagent was used.

A mechanism similar to the conventional cross-coupling reaction would operate [14] in the phenylation, *i.e.,* oxidative addition of **1** to palladium that generates [Cp(CO)_2_Fe–Pd–I], transmetalation with PhZnI•LiCl, and reductive elimination to yield **2a**.

### 2.2. Reactions of [CpFe(CO)_2_I] with Triphenylindium or -Aluminum

Triphenylindium, prepared from InCl_3_ and 3 equiv. of PhMgBr, could transfer the phenyl group to **1** under similar conditions (Table 3, entry 1) [15]. The amount of Ph_3_In could be reduced to 0.50 equiv (entry 2). However, the yield of **2a** was modest when 0.33 equiv of Ph_3_In was used (entry 3). Thus, two of the three phenyl groups on indium would be efficiently transferred [16]. The reaction of **1** with triphenylaluminum prepared from AlCl_3_ and 3 equiv of PhMgBr afforded **2a** sluggishly (entry 4).

**Table 3:**
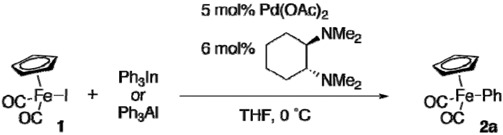
Palladium-Catalyzed Phenylation of [CpFe(CO)_2_I] with Triphenylindium or -Aluminum.

Entry	Phenylating Agent	time /h	NMR yield /%
1	Ph_3_In (1.5 equiv)	1	92
2	Ph_3_In (0.50 equiv)	7	83
3	Ph_3_In (0.33 equiv)	3	56
4	Ph_3_Al (1.5 equiv)	2	22

### 2.3. Reactions of [CpFe(CO)_2_I] with Arylboronic Acids 

Initially, a number of attempts to perform Suzuki-type arylation of **1** with phenylboronic acid failed to afford the corresponding aryliron **2a** when various phosphine ligands, amine ligands and bases were screened. We then found that bulky *N*-heterocyclic carbenes were good ligands. Especially, a combination of a palladium complex PEPPSI and cesium carbonate proved to provide marked improvement [17,18] (Table 4, entry 1). A variety of arylboronic acids underwent the arylation with high efficiency. In case that the arylation was not efficient enough, the addition of copper(I) iodide promoted the reactions (entries 5, 9, 11, 12, 14) [19,20,21,22]. We assume that arylcopper species generated from CuI and arylboronic acids would undergo more efficient transmetalation with the iodopalladium intermediate [23,24]. Notably, the combination of PEPPSI and copper(I) iodide allowed the synthesis of **2k** (entry 12), which could not be prepared from the corresponding arylzinc reagent. Styrene derivative **2r** was prepared from 4-vinylphenylboronic acid in high yield (entries 13 and 14). Unfortunately, the reactions with arylboronic acids having a hydroxy or amino group were sluggish (entries 15 and 16).

**Table 4:**
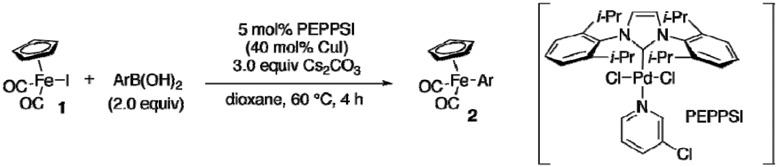
Palladium-Catalyzed Arylation of [CpFe(CO)_2_I] with Arylboronic Acids.

Entry	Ar	CuI /mol%	2	Isolated yield /%
1	Ph	0	**2a**	88
2	1-naphthyl	0	**2b**	82
3	2-MeC_6_H_4_	0	**2c**	76
4	4-MeOC_6_H_4_	0	**2d**	68
5	4-MeOC_6_H_4_	40	**2d**	82
6	4-CF_3_C_6_H_4_	0	**2o**	80
7	4-MeC_6_H_4_	0	**2p**	79
8	4-MeOCH_2_C_6_H_4_	0	**2q**	73
9	4-MeOCH_2_C_6_H_4_	40	**2q**	86
10	4-EtOC(=O)C_6_H_4_	0	**2i**	67
11	4-EtOC(=O)C_6_H_4_	40	**2i**	87
12	2-EtOC(=O)C_6_H_4_	40	**2k**	75
13	4-CH_2_=CHC_6_H_4_	0	**2r**	72
14	4-CH_2_=CHC_6_H_4_	40	**2r**	83
15	4-HOCH_2_C_6_H_4_	0	**2s**	0
16	3-H_2_NC_6_H_4_	0	**2t**	20

### 2.4. Transformation of [CpFe(CO)_2_Ar]

Previously, we developed oxidative methoxycarbonylation of [CpFe(CO)_2_(4-biphenylyl)] to afford methyl 4-biphenylcarboxylate [11,25,26,27]. The methoxycarbonylation proved to be applicable to functionalized [CpFe(CO)_2_Ar] prepared by the present method, leaving the functional groups untouched (Table 5). The yields of esters are good to excellent. The same set of the functionalized aryliron complexes also underwent photo-induced allylation (Table 6) [11]. The scope of the allylation is wide, and allylated products **5** were obtained in high yields.

**Table 5:**
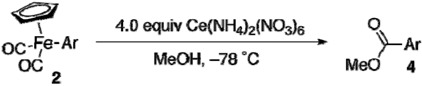
Oxidative Methoxycarbonylation of Functionalized Aryliron Complexes.

Entry	2	Time /h	4	Yield /%
1	**2d**	0.5	**4a**	45
2	**2g**	1	**4b**	61
3	**2h**	4.5	**4c**	59
4	**2i**	2	**4d**	91

**Scheme 1:**
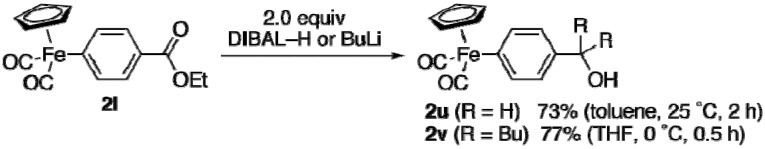


Treatment of **2i** with diisobutylaluminum hydride (DIBAL-H) in toluene or butyllithium in THF afforded benzylic alcohol **2u** or **2v**, respectively (Scheme 1). Nucleophilic attack to the ester moiety proceeded exclusively with the CpFe(CO)_2_ moiety untouched.

(Vinylphenyl)iron **2r** underwent ruthenium-catalyzed metathesis to expand the diversity of available aryliron complexes (Table 7) [28,29]. Self-metathesis of **2r** afforded (*E*)-stilbene derivative **2w** in excellent yield (entry 1). A cross-metathesis reaction of **2r** with 3 equiv of ethyl acrylate proceeded smoothly (entry 2). A cross-metathesis reaction with 1-octene or allyltrimethylsilane required a large excess of the alkene to achieve reasonable efficiency (entries 3 and 4). All the reactions proceeded with exclusive *E* selectivity. The aryl­–iron bonds were tolerant under the metathesis conditions.

**Table 6:**
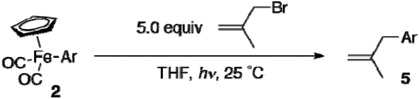
Allylation of Functionalized Aryliron Complexes under UV Irradiation.

Entry	2	Time /h	5	Yield /%
1	**2d**	2	**5a**	79
2	**2g**	1	**5b**	75
3	**2h**	2	**5c**	78
4	**2i**	15	**5d**	71

**Table 7:**
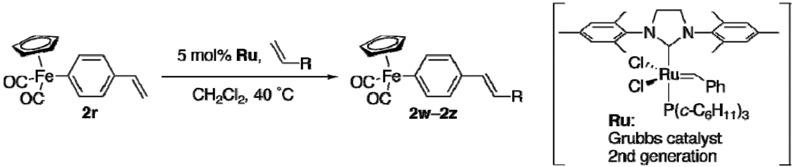
Ruthenium-Catalyzed Metathesis of **2r**.

Entry	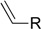	Amt. of 1-alkene /equiv	Time /h	2	Yield /%
1	**2r**	–	8	**2w**	91
2	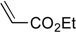	3	6	**2x**	79
3	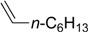	30	8	**2y**	76
4	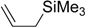	10	15	**2z**	33 (66^a^)

^a^ Yield determined by ^1^H-NMR analysis of the crude mixture.

## 3. Experimental Section

### 3.1. Instrumentation

^1^H-NMR (500 and 300 MHz) and ^13^C-NMR (125.7 and 75.3 MHz) spectra were taken on a Varian UNITY INOVA 500 spectrometer or a Varian GEMINI 300 spectrometer, using CDCl_3_ as solvent with tetramethylsilane as an internal standard. Chemical shifts (δ) are in parts per million relative to tetramethylsilane at 0.00 ppm for ^1^H and relative to CDCl_3_ at 77.2 ppm for ^13^C, unless otherwise noted. IR spectra were determined on a JASCO IR-810 spectrometer. TLC analyses were performed on commercial glass plates bearing a 0.25-mm layer of Merck Silica gel 60F_254_. Silica gel (Wakogel 200 mesh) was used for column chromatography. Mass spectra (EI unless otherwise noted) were determined on a JEOL Mstation 700 spectrometer. The elemental analyses were carried out at the Elemental Analysis Center of Kyoto University. Photochemical reactions were conducted with a 100-W high-pressure mercury lamp (SEN LIGHTS Corporation HB100P-1).

### 3.2. Chemicals

Unless otherwise noted, materials obtained from commercial suppliers were used without further purification. THF was purchased from Kanto Chemical Co., stored under argon, and used as-is. Dioxane was obtained from Wako Pure Chemicals Co., and stored over slices of sodium. Palladium acetate, cesium carbonate, ceric ammonium nitrate, and copper(I) iodide were obtained from Wako Pure Chemicals Co. PEPPSI and 2nd generation Grubbs catalyst were purchased from Aldrich. Arylzinc iodide-lithium chloride complexes were prepared according to the literature [13] and stored under argon. [CpFe(CO)_2_I] was prepared according to the literature [11].

*Typical Procedure for Arylation with Arylzinc Reagents (Table 2, entry 1):* THF (1.0 mL) was placed in a 20-mL reaction flask under argon. [CpFe(CO)_2_I] (**1**, 152 mg, 0.50 mmol), palladium acetate (0.050 M THF solution, 0.025 mL, 0.0013 mmol), *trans*-*N*,*N*,*N’*,*N’*-tetramethyl-1,2-cyclohexane-diamine (0.050 M THF solution, 0.050 mL, 0.0025 mmol), and phenylzinc iodide–lithium chloride complex (0.66 M THF solution, 1.14 mL, 0.75 mmol) were sequentially added at 0 °C. After the mixture was stirred for 15 min, a saturated ammonium chloride solution (1 mL) was added, and the product was extracted with ethyl acetate (10 mL × 3). Combined organic layer was passed through a pad of anhydrous sodium sulfate/Florisil and concentrated. ^1^H-NMR analysis of the crude product by using 1,1,2,2-tetrabromoethane as an internal standard indicated that **2a** was quantitatively formed. The crude oil was purified in air on silica gel by using carbon disulfide as an eluent to yield **2a** (114 mg, 0.45 mmol, 90%).

*Procedure for Phenylation with Triphenylindium (Table 3, entry 1):* Indium trichloride (1.55 g, 7.0 mmol) and THF (2 mL) were placed in a 20-mL reaction flask under argon. Phenylmagnesium bromide (1.03 M THF solution, 20.4 mL, 21 mmol) was added at 0 °C. The resulting mixture was stirred at ambient temperature overnight to prepare a THF solution of triphenylindium (0.31 M). THF (0.60 mL) was placed in another 20-mL reaction flask under argon. [CpFe(CO)_2_I] (**1**, 91 mg, 0.30 mmol), palladium acetate (3.4 mg, 0.015 mmol), *trans*-*N*,*N*,*N’*,*N’*-tetramethyl-1,2-cyclohexanediamine (3.6 mg, 0.018 mmol), and a THF solution of triphenylindium (0.31 M THF solution, 1.4 mL, 0.45 mmol) were sequentially added at 0 °C. After the mixture was stirred for 1 h, a saturated ammonium chloride solution (0.6 mL) was added, and the product was extracted with ethyl acetate (10 mL × 3). Combined organic layer was passed through a pad of anhydrous sodium sulfate/Florisil and concentrated. ^1^H- NMR analysis of the crude product by using 1,1,2,2-tetrabromoethane as an internal standard indicated that **2a** was obtained in 92% yield. Chromatographic purification using carbon disulfide as an eluent yielded **2a** (66 mg, 0.26 mmol, 87%).

*Typical Procedure for Arylation with Arylboronic Acids (Table 4, entry 1):* [CpFe(CO)_2_I] (**1**, 152 mg, 0.50 mmol), phenylboronic acid (122 mg, 1.0 mmol), PEPPSI (17 mg, 0.025 mmol), and cesium carbonate (489 mg, 1.50 mmol) were placed in a 20-mL reaction flask under an atmosphere of argon. 1,4-Dioxane (1.5 mL) was then added, and the resulting mixture was stirred for 4 h at 60 °C. The reaction was quenched with a saturated ammonium chloride solution (1 mL). Extractive workup followed by silica gel column purification (eluent: carbon disulfide) afforded **2a** (104 mg, 0.41 mmol) in 88% yield.

*Procedure for Oxidative Methoxycarbonylation Reactions of [CpFe(CO)_2_Ar] with Ce(NH_4_)_2_(NO_3_)_6_ (Table 5, entry 4):* Diammonium cerium(IV) nitrate (658 mg, 1.2 mmol) and methanol (6.0 mL) were added in a 50-mL reaction flask under argon. The mixture was cooled to –78 °C and then dicarbonylcyclopentadienyl(4-ethoxycarbonylphenyl)iron (**2i**, 98 mg, 0.30 mmol) in methanol (4.0 mL) was added slowly over 1 min. After being stirred for 2 h at –78 °C, the reaction mixture was quenched with aqueous solution of sodium thiosulfate and sodium bicarbonate. The products were extracted with diethyl ether (20 mL × 3). The combined organic layer was passed through pads of Florisil and sodium sulfate and concentrated. Silica gel column purification (eluent: hexane/ethyl acetate = 10:1) of the crude product provided ethyl methyl terephthalate (**4d**, 48 mg, 0.27 mmol, 91% yield).

*Procedure for Photo-induced Allylation Reactions of [CpFe(CO)_2_Ar] (Table 6, entry 4):* Dicarbonylcyclopentadienyl(4-ethoxycarbonylphenyl)iron (**2i**, 98 mg, 0.30 mmol), 3-bromo-2-methyl-propene (203 mg, 1.5 mmol), and THF (1.5 mL) were sequentially added in a quartz tube under argon. The reaction mixture was irradiated by a UV lamp at 25 °C and stirred for 2 h with irradiation. The distance between the reaction flask and the UV lamp was 2 cm. After irradiation, the mixture was filtered through a pad of Florisil, and the filtrate was concentrated. Silica gel column purification (eluent: hexane/ethyl acetate = 10:1) provided ethyl 4-(2-methyl-2-propenyl)benzoate (**5d**, 44 mg, 0.21 mmol, 71% yield).

*DIBAL-Reduction of* **2i***:* Aryliron **2i** (98 mg, 0.30 mmol) was placed in a reaction flask under argon. Toluene (1.5 mL) and DIBAL-H (1.0 M in hexane, 0.60 mL, 0.60 mmol) were sequentially added at 25 °C. After the mixture was stirred for 2 h, a 30% Rochelle salt solution (5 mL) was added slowly at 0 °C. The resulting mixture was stirred overnight. Extractive workup followed by silica gel column purification (eluent: CS_2_/CHCl_3_ = 1:1 to CHCl_3_ only) provided **2u** (62 mg, 0.22 mmol, 73%).

*Reaction of* **2i** *with Butyllithium:* Aryliron **2i** (98 mg, 0.30 mmol) and THF (1.0 mL) were placed in a reaction flask under argon. Butyllithium (1.66 M in hexane, 0.36 mL, 0.60 mmol) was added dropwise at 0 °C. After the mixture was stirred for 30 min, a saturated ammonium chloride solution (5 mL) was added slowly at 0 °C. Extractive workup followed by silica gel column purification (eluent: CS_2_/CHCl_3_ = 1:1) provided **2v** (92 mg, 0.23 mmol, 77%).

*Metathesis Reactions of* **2r***:* (Vinylphenyl)iron **2r** (84 mg, 0.30 mmol), dichloromethane (1.0 mL), and ethyl acrylate (0.098 mL, 0.90 mmol) were sequentially added in a reaction flask under argon. 2nd Generation Grubbs catalyst (13 mg, 0.015 mmol) was then added. After the mixture was stirred for 6 h at 40 °C, the mixture was passed through a pad of Florisil. The filtrate was concentrated *in vacuo*. Silica gel column purification (eluent: CS_2_/CHCl_3_ = 2:1) provided **2x** (85 mg, 0.24 mmol, 79%).

Characterization data for arylirons **2** [11,12], **4a** [30], **4b** [31], **4c** [31], **4d** [32], **5a** [33], **5b** [34], **5c** [35], and **5d** [36] were available in the literature. That of other compounds is given below.

*Dicarbonylcyclopentadienyl(3-trifluoromethylphenyl)iron* (**2e**): IR (neat) 2016, 1966, 1311, 1183, 1160, 1121, 1096, 1081, 1047, 790, 703, 676, 637, 621 cm^-1^; ^1^H-NMR δ = 4.89 (s, 5H), 7.05 (t, *J* = 7.5 Hz, 1H), 7.17 (d, *J* = 7.5 Hz, 1H), 7.64 (d, *J* = 7.5 Hz, 1H), 7.68 (s, 1H); ^13^C-NMR δ = 86.00, 120.05 (q, *J* = 3.8 Hz), 124.86 (q, *J* = 273.8 Hz), 126.98, 129.17 (q, *J* = 30.2 Hz), 140.37 (q, *J* = 3.8 Hz), 148.28, 148.41 (q, *J* = 1.5 Hz), 215.66; Found: C, 52.48; H, 2.91%. Calcd. for C_14_H_9_F_3_FeO_2_: C, 52.21; H, 2.82%.

*Dicarbonylcyclopentadienyl(2-fluorophenyl)iron* (**2f**): IR (nujol) 2028, 1977, 1454, 1193, 1016, 834, 764, 710, 629 cm^-1^; ^1^H-NMR δ = 4.91 (s, 5H), 6.80–6.88 (m, 2H), 6.95–6.99 (m, 1H), 7.49–7.52 (m, 1H); ^13^C-NMR δ = 85.57, 113.79 (d, *J* = 30.7 Hz), 123.86, 125.26 (d, *J* = 7.7 Hz), 127.99, 128.30, 146.73 (d, *J* = 13.0 Hz), 169.62 (d, *J* = 230.5 Hz) , 215.49; Found: C, 57.61; H, 3.41%. Calcd. for C_13_H_9_FFeO_2_: C, 57.39; H, 3.33%; m.p.: 57–58 °C.

*Dicarbonyl(4-cyanophenyl)cyclopentadienyliron* (**2h**): IR (nujol) 2225, 2014, 1964, 1942, 1920, 1575, 1462, 1418, 1042, 1010, 848, 818, 717 cm^-1^; ^1^H-NMR δ = 4.89 (s, 5H), 7.16 (d, *J* = 8.0 Hz, 2H), 7.61 (d, *J* = 8.0 Hz, 2H); ^13^C-NMR δ = 86.05, 106.54, 120.32, 129.13, 145.47, 161.07, 215.21; Found: C, 60.22; H, 3.24%. Calcd. for C_14_H_9_FeNO_2_: C, 60.25; H, 3.25%; m.p.: 100–101 °C.

*Dicarbonylcyclopentadienyl(4-ethoxycarbonylphenyl)iron* (**2i**): IR (nujol) 2023, 1949, 1708, 1576, 1454, 1283, 1124, 1008, 758 cm^-1^; ^1^H-NMR δ = 1.36 (t, *J* = 7.0 Hz, 3H), 4.33 (q, *J* = 7.0 Hz, 2H), 4.88 (s, 5H), 7.58 (s, 4H); ^13^C-NMR δ = 14.59, 60.55, 86.04, 125.76, 127.24, 144.96, 159.28, 167.97, 215.61; Found: C, 58.99; H, 4.43%. Calcd. for C_16_H_14_FeO_4_: C, 58.93; H, 4.33%; m.p.: 104–106 °C.

*Dicarbonylcyclopentadienyl(3-ethoxycarbonylphenyl)iron* (**2j**): IR (nujol) 2015, 1962, 1944, 1703, 1456, 1367, 1253, 1110, 751 cm^-1^; ^1^H-NMR δ =1.38 (t, *J* = 7.5 Hz, 3H), 4.35 (q, *J* = 7.5 Hz, 2H), 4.89 (s, 5H), 7.03 (t, *J* = 7.5 Hz, 1H), 7.58–7.60 (m, 1H), 7.65–7.67 (m, 1H), 8.12–8.13 (m, 1H); ^13^C-NMR δ = 14.56, 60.80, 86.00, 124.53, 127.03, 129.16, 145.36, 146.63, 149.66, 167.71, 215.82; Found: C, 58.79; H, 4.45%. Calcd. for C_16_H_14_FeO_4_: C, 58.93; H, 4.33%; m.p.: 67–68 °C.

*Dicarbonylcyclopentadienyl(2-ethoxycarbonylphenyl)iron* (**2k**): IR (nujol) 2020, 1969, 1945, 1707, 1456, 1377 cm^-1^; ^1^H-NMR δ = 1.37 (t, *J* = 7.0 Hz, 3H), 4.32 (q, *J* = 7.0 Hz, 2H), 4.94 (s, 5H), 6.97–6.99 (m, 2H), 7.44–7.46 (m, 1H), 7.72–7.74 (m, 1H); ^13^C-NMR δ = 14.48, 60.81, 86.85, 122.95, 128.10, 129.56, 146.55, 147.63, 147.70, 172.04, 215.62; Found: C, 58.78; H, 4.39%. Calcd. for C_16_H_14_FeO_4_: C, 58.93; H, 4.33%. m.p.: 64–65 °C.

*Dicarbonylcyclopentadienyl(2-thienyl)iron* (**2m**): IR (neat) 3104, 2854, 2026, 1966, 1420, 1391, 1317, 905, 826, 808, 682 cm^-1^; ^1^H-NMR δ = 4.98 (s, 5H), 6.83 (d, *J* = 3.0 Hz, 1H), 7.08 (dd, *J* = 3.0, 4.5 Hz, 1H), 7.45 (d, *J* = 4.5 Hz, 1H); ^13^C-NMR δ = 85.89, 128.89, 131.31, 134.68, 138.23, 214.72; Found: C, 50.55; H, 3.23%. Calcd. for C_11_H_8_FeO_2_S: C, 50.80; H, 3.10%.

*Dicarbonylcyclopentadienyl(4-trifluoromethylphenyl)iron* (**2o**): IR (nujol) 2007, 1957, 1588, 1325, 1158, 1072, 1008, 820, 631, 605 cm^–1^; ^1^H-NMR δ = 4.88 (s, 5H), 7.17 (d, *J* = 7.5 Hz, 2H), 7.59 (d, *J* = 7.5 Hz, 2H); ^13^C NMR δ = 86.01, 123.08 (q, *J* = 3.8 Hz), 125.01 (q, *J* = 272.3 Hz), 125.71 (q, *J* = 32.3 Hz), 144.94, 155.28 (q, *J* = 1.4 Hz), 215.63; Found: C, 52.07; H, 2.97%. Calcd. for C_14_H_9_F_3_FeO_2_: C, 52.21; H, 2.82%; m.p.: 72–73 °C.

*Dicarbonylcyclopentadienyl(4-methoxymethylphenyl)iron* (**2q**): IR (neat) 2920, 2012, 1955, 1480, 1187, 1096, 1042, 1007, 832, 797, 632 cm^-1^; ^1^H-NMR δ = 3.36 (s, 3H), 4.34 (s, 2H), 4.86 (s, 5H), 6.96 (d, *J* = 7.2 Hz, 2H), 7.43 (d, *J* = 7.2 Hz, 2H); ^13^C-NMR δ = 58.18, 75.02, 85.95, 127.50, 132.88, 145.04, 145.24, 216.19; Found: C, 60.69; H, 4.91%. Calcd. for C_15_H_14_FeO_3_: C, 60.43; H, 4.73%.

*Dicarbonylcyclopentadienyl(4-hydroxymethylphenyl)iron* (**2u**):IR (nujol) 3450, 2007, 1952, 1005 cm^‑1^; ^1^H-NMR δ = 1.48 (bs, 1H), 4.57 (d, *J* = 5.4 Hz, 2H), 4.87 (s, 5H), 7.00 (d, *J* = 7.5 Hz, 2H), 7.46 (d, *J* = 7.5 Hz, 2H); ^13^C-NMR δ = 65.68, 85.96, 126.75, 135.78, 145.24, 145.61, 216.12; Found: C, 58.97; H, 4.29%. Calcd. for C_14_H_12_FeO_3_: C, 59.19; H, 4.26%; m.p.: 92–93 °C.

*Dicarbonylcyclopentadienyl[4-(1-butyl-1-hydroxypentyl)phenyl]iron* (**2v**): IR (neat) 3480, 2935, 2862, 2016, 1961, 1684, 1456, 1003, 815 cm^-1^; ^1^H-NMR δ = 0.85 (t, *J* = 7.2 Hz, 6H), 1.10–1.29 (m, 8H), 1.61 (s, 1H), 1.72–1.77 (m, 4H), 4.86 (s, 5H), 6.96 (d, *J* = 8.1 Hz, 2H), 7.36 (d, *J* = 8.1 Hz, 2H); ^13^C-NMR δ = 14.27, 23.35, 25.95, 42.47, 76.72, 85.95, 124.78, 141.52, 141.93, 144.51, 216.40; Found: C, 66.91; H, 7.07%. Calcd. for C_22_H_28_FeO_3_: C, 66.68; H, 7.12%.

## 4. Conclusions

The palladium-catalyzed arylation of [CpFe(CO)_2_I] with arylzinc or arylboron reagents offers an efficient method for the synthesis of various functionalized iron complexes. Triphenylindium transfers the phenyl groups under palladium catalysis to arylate [CpFe(CO)_2_I]. The functionalized aryliron complexes [CpFe(CO)_2_Ar] undergo carbon-carbon bond formations with cleaving the carbon-iron bonds as well as functional group transformations without cleaving the carbon-iron bonds. The iron complexes thus synthesized can find many applications in material chemistry as well as in organic synthesis.
